# Validation of perceived physical fatigability using the simplified-Chinese version of the Pittsburgh Fatigability Scale

**DOI:** 10.1186/s12877-021-02275-x

**Published:** 2021-05-26

**Authors:** Yixin Hu, Hangming Zhang, Weihao Xu, Ming Zhao, Juan Liu, Linna Wu, Lin Zou, Jing Zuo, Yunxia Liu, Li Fan, Woei-Nan Bair, Yujia (Susanna) Qiao, Nancy W. Glynn

**Affiliations:** 1 Geriatric Health Care Department of The Second Medical Center & National Clinical Research Center for Geriatric Diseases, Chinese PLA General Hospital, Beijing, China; 2 Department of Orthopedics, Peking University Shougang Hospital, Beijing, China; 3 Outpatient Department, Haidian 37th Ex-Cadre Rest and Recuperation Center, Beijing, People’s Republic of China; 4 Geriatric Department of Beijing North Hospital of Ordnance Industry, Beijing, China; 5 Geriatric Emergency Department of The Second Medical Center & National Clinical Research Center for Geriatric Diseases, Chinese PLA General Hospital, Beijing, China; 6 Department of Physical Therapy, University of the Sciences in Philadelphia, Philadelphia, PA USA; 7 Department of Epidemiology, Center for Aging and Population Health, Graduate School of Public Health, University of Pittsburgh, Pittsburgh, PA USA

**Keywords:** Older adults, Fatigue, Validity, Physical performance, ADL

## Abstract

**Background:**

The Pittsburgh Fatigability Scale (PFS) was developed to capture fatigue and demand in a single tool, filling a gap that no validated questionnaire existed to measure perceived fatigability. Since fatigability is a more sensitive measure of a person’s susceptibility to fatigue, we validated the simplified-Chinese version of the PFS among Chinese community-dwelling older adults.

**Methods:**

This cross-sectional study was conducted in an urban community in Beijing between November 2018 and July 2019. The PFS was translated into simplified-Chinese by the translation, retro-translation method. Internal consistency of the Physical subscale of the PFS was evaluated by Cronbach’s alpha. Convergent validity and discriminant validity were evaluated against physical performance measures (i.e., Short Physical Performance Battery & Timed Up and Go Test) and daily living performance (i.e., Barthel Index & Instrumental activity of daily living).

**Results:**

Our study included 457 participants, including 182 men (39.8%) and 275 women (60.2%). The age range of the included participants was 61–96 years (mean = 84.8 years, SD = 5.8 years). The simplified-Chinese version of PFS Physical scores showed strong internal consistency (Cronbach’s alpha = 0.81). Higher PFS Physical scores were associated with worse physical performance, and daily living performance (|correlation coefficient| range: 0.36–0.56, *p* < .001). Age- and sex-adjusted PFS Physical scores had moderate to good overall discrimination for correctly classifying people by their physical performance and daily living performance (AUCs range 0.70–0.87, *p* < .001).

**Conclusions:**

The PFS simplified-Chinese version is a valid instrument to assess perceived physical fatigability in Chinese-speaking older adults with good convergent validity. Thus, the PFS, with low cost and greater feasibility, is a desired tool to measure fatigability in large population studies.

## Background

In developing countries, people aged 60 years and older represent the fast-growing proportion and absolute number [1]. The 2010 China Census revealed that 13.3% (i.e., 177.6 million) of the total population was represented by older persons [2]. Fatigability is a common and distressing self-perceived symptom while performing usual mental and physical activities, especially among older adults, and strongly associated with adverse health-related events [3]. Perceived fatigability measures an individual’s whole-body tiredness anchored to standardized activity of a specific intensity and duration, which may help to overcome the limitation of current fatigue scales by providing a less-biased and more sensitive measure [3–8].

Since there was no questionnaire-based tool to measure perceived fatigability, Glynn et al. developed the Pittsburgh Fatigability Scale (PFS) in English, a 10-item validated tool evaluating both physical and mental fatigability in separate subscales [6]. The PFS Physical subscale has good convergent validity against performance measures of mobility, physical function and fitness, as well as good concurrent validity against performance-based measures of fatigability [6]. Greater perceived physical fatigability using the PFS has been shown to be higher in women than men, associated with poorer mobility, meaningful decline in physical function, and other important health indicators [3, 4, 7, 9, 10].

Short Physical Performance Battery (SPPB), walking speed and timed “Up & Go” test (TUG) [11] are commonly used objective measures to explore clinically meaningful change in physical performance in clinical trials [12]. And it is advocated by International Conference of Frailty and Sarcopenia Research (ICFSR) Task Force to incorporate patient reported outcome measures (PROMs) with subjective measures to provide supplementary information to access the physical function of patients. PFS was one of a good patients reported outcome measures, especially for those unable to perform particular activities. Given the importance of measuring fatigability in older adults [8], and with a lack of a validated tool in China, it is important to evaluate the validity of the PFS in Chinese community-dwelling older adults against different physical function assessment tools.

This study aimed to validate the simplified-Chinese version of the PFS Physical subscale by assessing convergent validity against physical performance and daily living performance among Chinese older adults.

## Methods

### Data and study participants

The present cross-sectional study of older adults from an urban retirement community was performed in Beijing between November 2018 and July 2019. It was the first wave of an ongoing longitudinal study. This study was approved by the Research Ethics Committee of Chinese PLA General Hospital (Ethic number: S2018–102–02) and has been registered in Chinese Clinical Trial Register (ChiCTR1900022576). All participants signed the written informed consent.

**Fig. 1:**
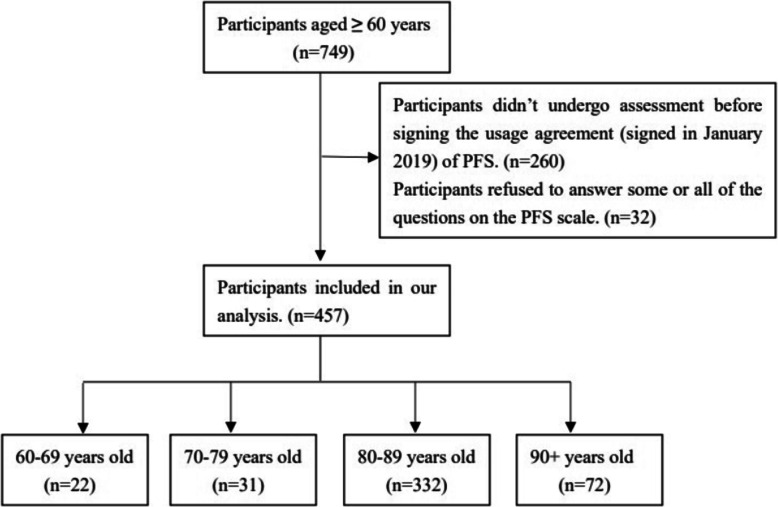
The analysis sample selection process and age distribution of this study

We recruited participants from all senior citizens living in the retirement community. The inclusion criteria were: 1) age 60 years or over and 2) voluntary participation in this study. The exclusion criteria were: (1) unable or refuse to perform Timed Up and Go Test (TUG) test or gait speed test or refused to take the handgrip strength; (2) had severe cognitive impairment or dementia diagnosed by neurologist; (3) had severe hearing problem; (4) had an implanted electronic device or orthopedic metal implantations; and (5) had terminal cancer. All of the interviewers were well-trained before the study inception. Participants’ data were collected by face-to-face interview and real-time physical and cognitive measurements at the community medical center or their home. A total of 749 participants were recruited from the community, and 457 participants with PFS were included in our analysis (Fig. 1). Participants would get a multidisciplinary health instruction manual, and no other economic gains.

### Linguistic translation of the simplified-Chinese version of PFS

The validated method of translation, Retro-translation of the Pittsburgh Fatigability Scale was followed to obtain the final simplified-Chinese version of the PFS [13]. Two Chinese-speaking researchers independently translated the PFS into simplified-Chinese. Discrepancies were discussed and an agreement was reached by consensus. We administered the simplified-Chinese version of the PFS to two Chinese older community-dwellers to evaluate the proper understanding of items used in the scale. Then, the simplified-Chinese version of the PFS was retro-translated into English by a bilingual researcher and then checked for accuracy by another independent bilingual individual. For culturally relevancy some example activities (dusting, straightening up, baking; raking; aerobic machines, Zumba) from the original PFS were removed and a new example, Ping-Pong was added to the high-intensity activity item. We have a signed copyright agreement with the University of Pittsburgh that provided permission to use the Simplified Chinese Version of PFS for this study. The final simplified-Chinese version of the PFS is available upon request by the developer: https://publichealth.pitt.edu/epidemiology/research-practice/faculty-research/pittsburgh-fatigability-scale.

### Measurement of perceived physical fatigability

The 10-item Pittsburgh Fatigability Scale was used to assess perceived physical fatigability. Participants were asked to rate their level of physical tiredness that they expected or imagined they would feel after completing each activity on a scale from 0 (no fatigue) to 5 (extreme fatigue). These activities ranged in intensity from low (e.g. watching TV for 2 h) to high (e.g. high-intensity activity for 30 min). Responses to each item were summed to create PFS Physical scores ranging from 0 to 50 (higher scores = greater perceived fatigability). A PFS Physical score ≥ 15 denotes greater perceived physical fatigability [3, 7]. Additionally, PFS Physical scores were categorized into six groupings according to previously published work [3, 7], and then collapsed into three strata due to distribution of the sample to examine severity of perceived physical fatigability.

### Physical performance measures

The Short Physical Performance Battery (SPPB) includes three objective tests:1) two-timed 4-m walks at a usual pace; 2) rising 5 times from a seated position to an upright position as quickly as possible with the arms folded across the chest; and 3) three individual standing balance tests, including 3 progressively more difficult positions for 10s each: side-by-side stand, semi-tandem stand, and tandem stand [14]. Lower limb strength was measured by the total time of repeated chair stands. Each of the 3 tests was scored 0 (worst performance) to 4 (best performance). The three tests were summed, and a total SPPB score ranged from 0 to 12, with higher scores reflecting better physical function. Timed “Up & Go” test (TUG) asked participants to stand up from a standard chair, walk a distance of three meters at a normal pace, turn, walk back to the chair and sit down, and the time to complete the entire maneuver was recorded using a stopwatch (within 0.1 s) [11, 15]. Both SPPB and TUG were conducted in Chinese.

### Daily living performance

The Barthel Index was used to measure the Activities of Daily Living (ADL) [16] in Chinses version, ranging between 0 and 100 with lower scores indicating higher level of dependency, and subjects with score higher than 80 should be able to live independently. Instrumental Activity of Daily Living (IADL) in Chinses version was measured by the 8-item scale developed by Lawton and Brody [17]. IADL dependence was defined as self-reported inability to perform any of the following tasks independently: using a telephone, shopping, preparing food, doing laundry, administering medications, going places, housekeeping, or personal finances.

### Covariates

Covariates were measured from questionnaires which included age, sex, education level, marital status (married vs. others including divorce, widowed, and never married), smoking status, body mass index (BMI), nutritional status, and self-reported physician-diagnosed diseases including hypertension, coronary artery disease (CAD), chronic obstructive pulmonary diseases (COPD), diabetes mellitus, and chronic kidney disease (CKD).

### Statistical analyses

The descriptive statistics for continuous variables were by mean ± standard deviation and categorical variables were by N (%). Cronbach’s alpha was used to measure the internal consistency of the simplified-Chinese version of the PFS Physical scores. Commonly, a Cronbach’s alpha coefficient is preferably above 0.8, and 0.7 to 0.8 is considered acceptable [18].

Spearman’s rank-order correlations were used to examine the correlations of the PFS Physical scores with physical performance and daily living performance. The strength of correlations reflected via Spearman correlation coefficients [19]: |r| ≤ 0.3: low or weak correlation; |r| = 0.3–0.5: moderate correlation; and |r| ≥ 0.5: strong correlation.

When using the dichotomous measures of distinguishing different levels of physical performance and daily living performance, the least-square means and standard error adjusted for age and sex of the two groups were calculated. SPPB < 10 points indicated low SPPB, and slower gait speed was defined as gait speed < 1.0 m/sec, and poor lower limb strength was defined as the time to performed chair stand test ≥16.7 s or not able to do 5 stands [20]. The cut-off value of low mobility was TUG time ≥ 14.0 s, while the Barthel Index ≤80 indicated ADL dependence, and IADL ≤7 indicated IADL dependence. We made the physical performance and daily living performance as the state variable and used the PFS scores as the test variable. The discriminant validity of PFS score was assessed by the Area Under the Curve (AUC): AUC: 0.5–0.7, poor or low; AUC: 0.7–0.8, fair or moderate; and AUC: 0.8–1.0, good or excellent. All statistical analyses were performed using Statistical Package for Social Science (SPSS) software, version 22.

## Results

### Sample description

**Fig. 2:**
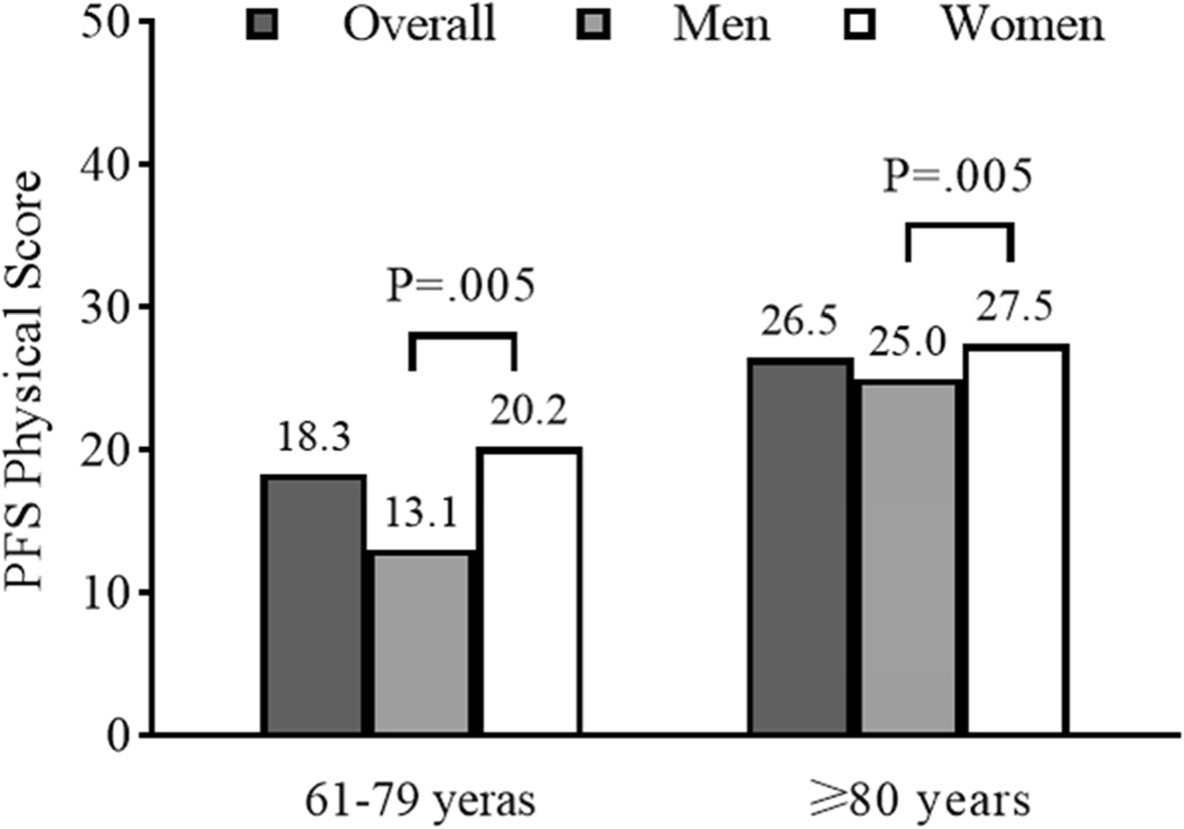
Mean PFS Physical scores by age strata and sex

The average age of the 457 participants was 84.5 ± 5.8 years, 60.2% women, 27.7% had lower educational status, and 61.3% were married. The average BMI of the participants was 24.0 ± 3.5 kg/m^2^. Mean PFS Physical score was 25.5 ± 9 points, and 403 (88.2%) of them had a PFS score ≥ 15, and 271 (59.3%) of them ≥25 (Table 1). Overall, women had higher PFS Physical scores than men, 26.4 ± 8.4 versus 24.1 ± 9.4, respectively, *P* = .007 (Fig. 2). PFS Physical scores were distributed across the range of potential values, and no sign of ceiling or floor effects. The Cronbach’s alpha of the simplified-Chinese version of the PFS was 0.81, which showed good internal consistency.

**Table 1:** Participant Characteristics of the Simplified-Chinese version PFS Validation Sample by Perceived Physical Fatigability Severity Strata

Characteristics	Total sample ***N*** = 457	PFS Physical Score Severity Strata		
		Less Fatigability 0–14 *n* = 54	15–24 *n* = 132	Most Fatigability ***≥***25 *n*= 271
Age, years	84.8 ± 5.8	80.2 ± 8.7	84.5 ± 5.8	85.8 ± 4.5
≥ 80 years	404 (92.3)	35 (64.8)	116 (87.9)	253 (93.4)
Median (IQR)	86.0 (83.0–88.0)	83.0 (72.8–87.3)	85.5 (83.0–88.0)	86.0 (83.0–89.0)
Women	275 (60.2)	26 (48.1)	65 (49.2)	184 (67.9)
Married	280 (61.3)	35 (64.8)	93 (70.5)	152 (56.1)
Education years				
< 12 (Below high school)	127 (27.8)	16 (29.6)	25 (18.9)	86 (31.7)
= 12 (High school)	109 (23.9)	15 (27.8)	28 (21.2)	66 (24.4)
> 12 (Above high school)	221 (48.4)	23 (42.6)	79 (59.8)	119 (43.9)
Monthly income, RMB/ USD				
< 10,000/ 1549.4	201 (44.0)	23 (42.6)	51 (38.6)	127 (46.9)
≥ 10,000/ 1549.4	256 (56.0)	31 (57.4)	81 (61.4)	144 (53.1)
BMI, kg/m^2^	24.0 ± 3.5	24.3 ± 3.3	24.2 ± 3.1	23.9 ± 3.7
Smoking status				
Current	8 (1.8)	2 (3.7)	3 (2.3)	3 (1.1)
Previous ^a^	91 (19.9)	10 (18.5)	31 (23.5)	50 (18.5)
Never	358 (78.3)	42 (77.8)	98 (74.2)	218 (80.4)
MNA score, 0–14 range	12.9 ± 1.6	13.3 ± 1.0	13.2 ± 1.1	12.7 ± 1.8
≤ 11	64 (14.2)	4 (7.4)	12 (9.2)	48 (18.1)
Health Conditions				
Hypertension	320 (70.6)	35 (64.8)	88 (67.2)	197 (73.5)
CAD	227 (50.3)	23 (42.6)	60 (45.8)	144 (54.1)
COPD	82 (18.2)	3 (5.6)	27 (20.8)	52 (19.5)
Diabetes	132 (28.9)	10 (18.5)	33 (25.2)	89 (32.8)
CKD	45 (10.0)	6 (11.1)	12 (9.2)	27 (10.1)
Number of comorbidities				
0–1	54 (12.5)	20 (15.9)	24 (9.4)	10 (19.2)
2–4	218 (50.3)	62 (49.2)	124 (48.6)	32 (61.5)
≥ 5	161 (37.2)	44 (34.9)	107 (42)	10 (19.2)
Total SPPB score, 0–12 range	8.4 ± 2.8	10.3 ± 1.9	9.1 ± 2.3	7.5 ± 2.8
10–12	141 (39.9)	37 (71.2)	53 (48.6)	51 (26.6)
7–9	116 (32.9)	13 (25.0)	42 (38.5)	61 (31.8)
≤ 6	96 (27.2)	2 (3.8)	14 (12.8)	80 (41.7)
Gait speed, m/s	0.9 ± 0.3	1.1 ± 0.3	1.0 ± 0.2	0.8 ± 0.2
< 1 m/s	273 (67.9)	18 (34.0)	74 (57.8)	181 (81.9)
Chair stand test time, s	14.2 ± 4.9	12.5 ± 4.8	12.8 ± 3.1	15.8 ± 5.4
≥ 16.7 s or incapable ^b^	133 (35.2)	3 (5.9)	30 (28.6)	100 (45.0)
TUG time, s	12.9 ± 4.9	11.4 ± 5.4	12.1 ± 4.0	14.0 ± 5.3
≥ 14.0 s	112 (33.0)	10 (19.6)	33 (26.8)	69 (41.8)
ADL ≤ 80	48 (10.5)	1 (1.9)	0 (0.0)	47 (17.4)
IADL ≤ 7	284 (62.1)	18 (33.3)	67 (50.8)	199 (73.4)

Values are presented in mean ± standard deviation, N (%) and median (IQR)

*BMI* Body Mass Index, *MNA* Mini Nutritional Assessment, *CAD* Coronary Artery Disease, *COPD* Chronic Obstructive Pulmonary Disease, *CKD* Chronic Kidney Disease, *PFS* Pittsburgh Fatigability Scale, *SPPB* Short Physical Performance Battery, *TUG* Timed Up and Go Test, *ADL* Activities of Daily Living, *IADL* Instrumental Activities of Daily Living, *IQR* Interquartile Range

^a^ Defined as used to smoke and has quit smoking for more than 6 months

^b^ “Incapable” means the participants did not complete the 5 times chair stand test for physical reasons or safety reasons

### Convergent validity

Higher PFS Physical scores were correlated with both lower extremities. Specifically, for SPPB score (*r* = −0.51), gait speed (*r* = −0.55), the time to complete 5 chair stands (*r* = 0.40) and TUG test (*r* = 0.28). For daily living performance, higher PFS Physical scores were also associated with ADL (*r* = − 0.56) and IADL (*r* = − 0.48), all *p* < .001 (Table 2).

**Table 2:** Convergent Validity of the PFS Physical Scores -Simplified-Chinese version

Characteristics	Correlation Coefficient (r) ^**a**^ with the PFS Physical scores	***P***-Value
Total SPPB score	−0.51	< .001
Gait speed, m/s	−0.55	< .001
Chair stand test time, s	0.40	< .001
TUG, s	0.28	< .001
Barthel Index	−0.56	< .001
IADL	−0.48	< .001

*SPPB* Short Physical Performance Battery, *TUG* Timed Up and Go Test, *ADL* Activity of Daily Living, *IADL* Instrumental Activity of Daily Living

^**a**^ Spearman’s rank-order correlations

Age- and sex-adjusted PFS Physical scores were significantly higher in subgroups of worse physical and daily living performance, indicating greater perceived physical fatigability (differences 2.2 to 10.7 points, Table 3). Moreover, AUC values (Table 3) indicated PFS Physical scores had moderate to excellent overall discrimination for correctly classifying people by their physical and daily living performance status (AUCs: 0.70–0.87).

**Table 3:** Concurrent and convergent validity of the PFS Physical scores: Simplified-Chinese Version with physical performance and physical ability measures

Measures	LS means (SE) ^**a**^		Difference		AUC (95% CI)
	Yes	No	Mean (SE)	***P***-Value	
Low SPPB^b^	27.0 (3.4)	19.4 (3.0)	7.7 (0.4)	< .001	0.77 (0.72, 0.81)
Slower gait speed ^c^	26.9 (3.3)	19.0 (4.0)	7.8 (0.4)	< .001	0.76 (0.72, 0.80)
Poor lower limb strength^d^	26.8 (2.4)	24.6 (4.2)	2.3 (0.4)	< .001	0.78 (0.74, 0.82)
Low mobility^e^	25.7 (4.2)	21.7 (4.1)	4.3 (0,9)	< .001	0.77 (0.72, 0.81)
ADL dependence^f^	35.1 (5.7)	24.4 (3.9)	10.7 (0.6)	< .001	0.87 (0.84, 0.90)
IADL dependence^g^	28.1 (4.1)	21.3 (3.3)	6.8 (0.4)	< .001	0.81 (0.77, 0.84)

*LS means* Least Squares means, *SE* Standard Error, *AUC* Area Under the Curve (the different levels of physical performance and daily living performance were used as the state variable and the PFS Physical scores were used as the test variable directly), *SPPB* Short Physical Performance Battery, *ADL* Activities of Daily Living, *IADL* Instrumental Activities of Daily Living

^a^ Adjusted for age and sex

^b^ Defined as SPPB < 10 points

^c^ Defined as gait speed < 1.0 m/s

^d^ Defined as the time to performed chair stand test ≥ 16.7 s or not able to do 5 stands

^e^ Defined as Timed Up and Go Test time ≥ 14.0 s

^f^ Defined as Barthel Index ≤ 80

^g^ Defined as IADL ≤ 7

## Discussion

The present study was the first to translate the PFS into simplified-Chinese, modified according to cultural context and validated among mainland Chinese older adults. This study provided the evidence for the validity of the simplified-Chinese version of the PFS Physical subscale in Chinese older adults. Our findings indicated that PFS Physical scores were moderate to strongly associated with physical performance (SPPB, gait speed, TUG) and daily living performance (ADL and IADL). Greater perceived physical fatigability was significantly associated with worse physical and daily living performance. Overall, the simplified-Chinese version of the PFS exhibited moderate to excellent convergent validity and strong internal consistency.

The original English PFS has been translated into 12 languages, with several versions already validated in their associated populations. The English version of the PFS [6] presents high concurrent and convergent validity. Like the original and Spanish versions of the PFS, the simplified-Chinese version was able to distinguish those exhibiting high versus low fatigability against physical performance measures [6, 21]. Additionally, the English and Dutch versions of the PFS had good reliability for the Physical subscale with intraclass correlations ≥0.80 [6, 19]. The PFS Physical scores of our study were higher than others, likely because our population was older. In our population of older adults from Beijing, 88.2% of this population (mean age around 85 years) were categorized as having greater perceived physical fatigability comparable to the oldest-old (≥90 years) from the Long Life Family Study (LLFS), a generally healthier population enriched for exceptional longevity [10]. In the LLFS [10], mean PFS Physical scores of those 80–89 year and ≥ 90 years old were 19.1 and 28.6 points, respectively, indicating that our sample had greater fatigability, with a mean of 25.5 points. Similar to LaSorda et al. [10], we found that women had significantly higher PFS Physical scores than men.

ADL and Instrument ADLs are common questionnaires for assessment of disability, but are less sensitive for their “ceiling effect” [17, 22], especially for the individual’s healthy status modifications. Further, it is of importance to identify those with physical limitations in clinical settings, since they may benefit more from early interventions to reduce physical fatigability. Earlier functional limitations are commonly measured with the SPPB and its components [23]. Total SPPB score is a strong predictor of mobility disability and ADL disability [14]. Gait speed, which could be easily measured in clinical setting and communities, has been reported by many studies, and independently predicts several adverse health outcomes [24, 25]. In the present study, we found that the PFS not only had good concurrent and convergent validity against ADL and IADL, but also was positively associated with total SPPB score and gait speed. Our findings concur with findings from the Long Life Family Study [10] showing that having difficulty with ADLs (physical disability) was a risk factor associated with greater perceived physical fatigability. The current study also indicated that perceived physical fatigability was a risk factors associated with physical limitation in this very old Chinese group.

The PFS includes commonly performed activities by older adults ranging from low to high-intensity. Even persons who are unable to perform particular activities can complete the PFS by imaging their perception of tiredness, and the PFS Physical score has previously demonstrated high concurrent validity and strong convergent validity against both functional and physical performance measures in other countries and studies [6, 21]. Additionally, the PFS is a brief, simple tool which is easy and inexpensive to use, especially for large population-based studies. Moreover, using the PFS can better elucidate how fatigability fits in the disablement pathway and functional decline, and identify older adults at risk of dysfunction in clinical and research settings instead of using performance-based fatigability measures that some older adults are unable to do.

The strengths of this study include the relatively large sample size and the assessment of convergent validity of PFS with a wide variety of performance-based measurements and daily living performance. Further, the linguistic translation of the simplified-Chinese version of the PFS used the validated method of translation, retro-translation. A limitation of this study is that the sample came from one community of retired older adults living in city, limiting generalizability due to potential selection bias. The validity among those with severe hearing issues remained unknown and needs further research due to them being excluded. Since China is geographically large and includes many ethnic minorities, further validation studies should be conducted in rural areas of China and include a more diverse population.

## Conclusions

The PFS, simplified-Chinese version, is a brief and easy to use tool to measure perceived physical fatigability in Chinese older adults. The PFS Physical subscale has high concurrent and convergent validity against physical performance measurements and daily living performance. This is of great significance for improving the evaluation of fatigability impact and identifying older adults at risk of mobility limitation in clinical and research settings. This study also provides the possibility of using perceived physical fatigability as clinical indicator or predictor variable in further studies, as it is an important prognostic measure of phenotypic aging.

## Data Availability

The datasets used and analysed during the current study are available from the corresponding author on reasonable request.

## Abbreviations

BMI: Body Mass Index
MNA: Mini Nutritional Assessment
CAD: Coronary Artery Disease
COPD: Chronic Obstructive Pulmonary Disease
CKD: Chronic Kidney Disease
PFS: Pittsburg Fatigability Score
SPPB: Short Physical Performance Battery
TUG: Timed Up and Go Test
ADL: Activities of Daily Living
IADL: Instrumental Activities of Daily Living
IQR: Interquartile Range
LS means: Least Squares means
SE: Standard Error
AUC: Area Under the Curve
